# Spatial Epidemic Modelling in Social Networks

**DOI:** 10.1063/1.1985395

**Published:** 2005-06-21

**Authors:** Joana Margarida Simoes

**Affiliations:** Centre for Advanced Spatial Analysis, University College of London, UK; University of Aveiro; University of Aveiro; University of Aveiro; University of Aveiro; University of Aveiro

## Abstract

The spread of infectious diseases is highly influenced by the structure of the underlying social network. The target of this study is not the network of acquaintances, but the social mobility network: the daily movement of people between locations, in regions. It was already shown that this kind of network exhibits small world characteristics. The model developed is agent based (ABM) and comprehends a movement model and a infection model. In the movement model, some assumptions are made about its structure and the daily movement is decomposed into four types: neighborhood, intra region, inter region and random. The model is Geographical Information Systems (GIS) based, and uses real data to define its geometry. Because it is a vector model, some optimization techniques were used to increase its efficiency.

### INTRODUCTION

Since a long time, human Epidemics have interested scientists of several areas.

Once diseases spread amongst people, it is impossible to ignore the role of social networks in embedding this phenomenon. Therefore, the architecture and general topological features of the social network should be considered in the model.

In several studies it has been considered the network of acquaintances [1], which is important in diseases that require a close, prolonged contact. However, in highly contagious diseases, the infection may be passed by a short physical contact, and in this case it is more important to track the movement of individuals.

In this model, it was considered the social mobility network: the daily movement of individuals, which has been already referred in the literature as a complex network with a Small World behaviour [2].

In complex systems, the interaction among the constituents of the system, and the interaction between the system and its environment, are of such a nature that the system cannot be fully understood by simply analysing its components [3]. In this paper, it is described a simulation system using artificial agents integrated with Geographical Information Systems (GIS) that helps to understand the spatial and temporal behaviour of a epidemic phenomena.

The utility of spatially explicit agent oriented simulations is demonstrated by simulating alternative scenarios to compare differences in the spatial structure of the mobility network and the geographical distribution of individuals.

CP776, *Science of Complex Networks: From Biology to the Internet and WWW; CNET 2004,*
edited by J. F. F. Mendes, S. N. Dorogovtsev, A. Povolotsky, F. V. Abreu, and J. G. Oliveira
© 2005 American Institute of Physics 0-7354-0262-0/05/$22.50

### Conceptual Model

Agent based models (ABM) offer a great potential for studying human complex behaviour, interacting within a spatial framework [4].

Unlike what happens with Cellular Automata (CA), in ABM space is continuous and location is explicit, which means that individuals can be simulated, independent of the environment. This allows to specify rules focused on the individuals, and not on space.

The present model is inspired by a Site Exchange Cellular Automata [5], which considers two phases for each time step: movement and infection, assuming there is no virus transmission while the individual is moving. The movement rules and infection rules are determined by a movement model, and a infection model that are going to be described on the next paragraphs.

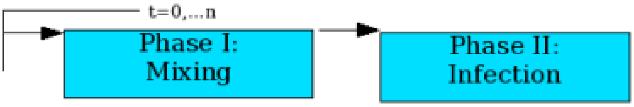

**FIGURE 1.** Two sub-phases of each step of the SECA.

### Movement Model

The domain of the model is divided into small subunits with a geographical relevance: the regions. This regions can have several definitions depending on the scale, but in this case it were consider the concelhos, according to the INE definition (administrative division code/1994 revision approved by the deliberation n 86 of 15/12/1994). This choice was motivated by data availability and also for keeping the simulations reasonably fast. However, the model should be run with smaller regions, once conceptually the region definition is closer to the city definition.

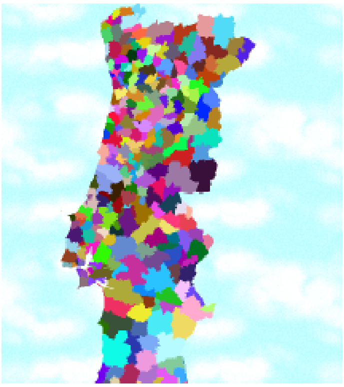

**FIGURE 2.** Model domain, with division in regions.

The movement rules try to emulate the daily movement of individuals according to the diversity of its activities (working, shopping, etc.). Based on these regions, four ranges of movement were considered: neighbourhood, intra region, inter region and small world.

Neighborhood is the random movement of a individual within its immediate neighborhood (in this case it was considered a radius, defined as a parameter). This stands for the motion of an individual within its street or neighborhood; it is considered that it is where it spends most of its time.

Intra region is the random movement inside a region. This stands for the movement of an individual inside a city: for instance for working or going to the cinema.

Inter region is the random movement of an individual on the neighbor regions. This represents the travel to nearby cities, for instance for shopping or visiting friends and family.

Finally, it was considered a total random movement named Small World (SW). This movement is a very tiny fraction of the total, and represents the situation that a small number of individuals have large range movements, which produce the shortcuts in the network, responsible for the SW phenomena.

Probabilities were attributed to each kind of movement, based on common sense of what are the most probable activities. This weights or probabilities should be based on real mobility data that were not available for this study, and therefore the results should be seen with caution.

On Fig. 3, it is shown a graphic with the probabilities attributed to each kind of movement.

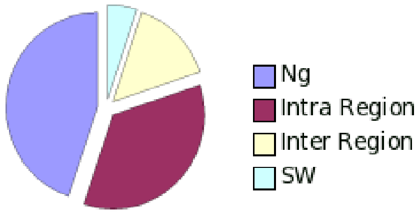

**FIGURE 3.** Proportions of each range of movement.

The update of the movement model is synchronous.

### Infection Model

The infection model considered was a SLIR (Susceptible-Latent-Infected-Removed).

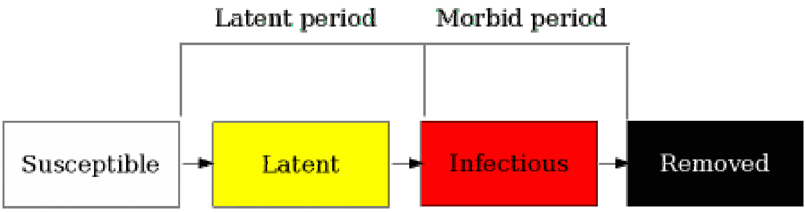

**FIGURE 4.** Different categories of the infection model.

The contagious (state change from susceptible to latent) occurs every time a susceptible meets a infectious within a certain radius. The state change from latent to infectious, and infectious to removed is only determined by time.

The contagious model is not specific for a disease, but it is flexible enough to be adjusted and fit the characteristics of a given disease.

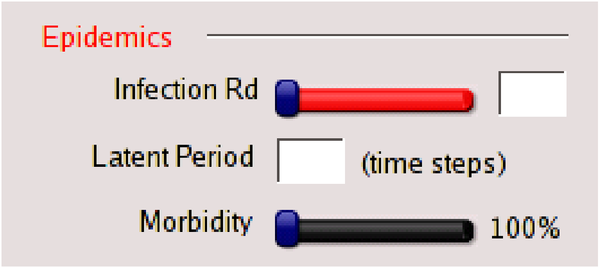

**FIGURE 5.** Infection parameters.

The update of the movement model is synchronous .

### IMPLEMENTATION OF THE MODEL

The model is vector based, considering points moving continuously over a polygon layer (Fig. 6).

The polygon layer reads the geographic information from a *shapefile* (the ESRI GIS format), using the *shapelib* API. The shapelib provides the programmer a structure with all the information contained in the *shapefile*, and in this way it is not necessary to program the low level access to the datafile (Fig. 7).

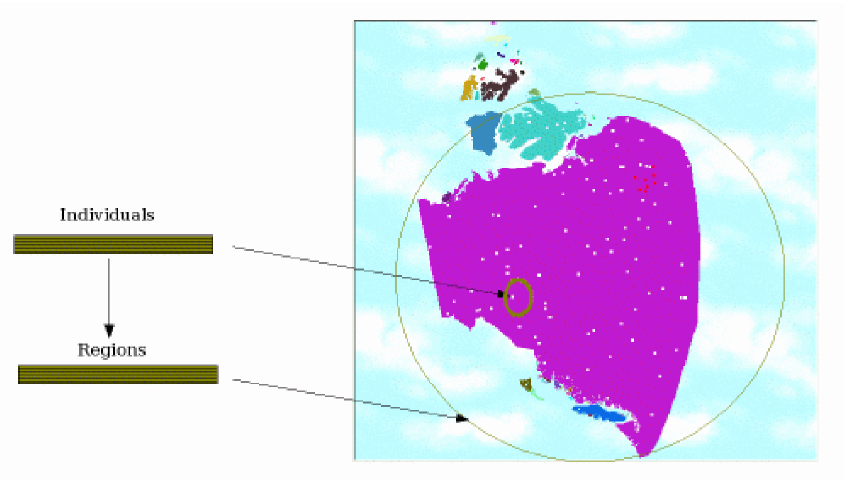

**FIGURE 6.** Layers considered in this model.

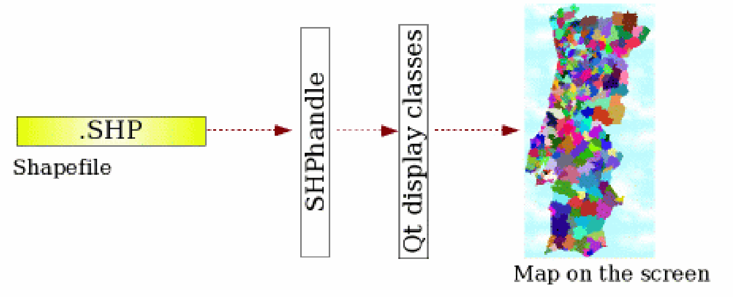

**FIGURE 7.** *Shapefiles* reading and display implementation details.

The advantage of reading these files is the possibility of displaying different geographical configurations.

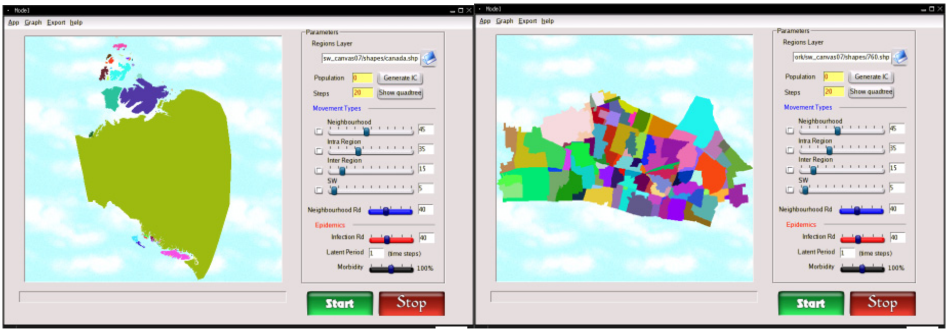

**FIGURE 8.** Different geographical domains and regions.

Several GIS functionalities were implemented (like buffers and zooms) and the algorithms were optimized taking into consideration the speed of the simulations. Once the operation of searching for features was intensively used, one of the efficiency algorithms developed was a search method: the quadtree.

A quadtree is a tree structure used to encode two-dimensional spaces. The image is recursively subdivided into sub quadrants. The quadtree is used for raster spaces, so in this case it was used a adaptation of the quadtree for vectorial spaces.

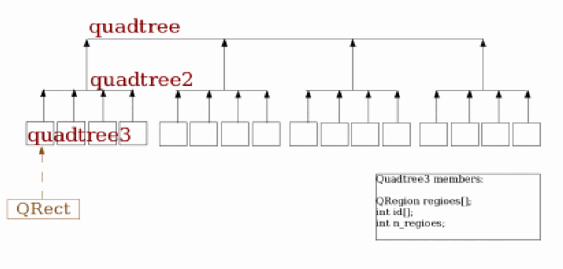

**FIGURE 9.** Structure of the vectorial quadtree implemented in this model.

## SENSITIVITY ANALYSIS

Structure of the vectorial quadtree implemented in this model.

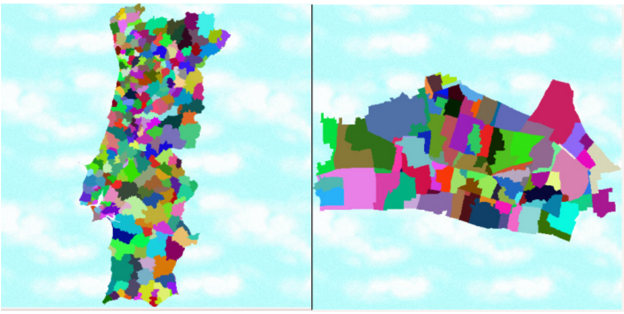

**FIGURE 10.** Simulations for shapefile A and B.

The sensitivity analysis of the movement model was performed by running simulations for exclusively one type of movement.

The domain of the model for these simulations was Portugal, with concelhos as regions.

The initial conditions are the population distribution of Census 91 (INE) and the reported cases of mumps in 1993, which were in the origin of a small epidemic in this country.

Although the simulations are agent based, the results are shown at the level of region, in order to be more perceptible. Darker shadings correspond to a greater amount of infected and removed individuals in the region.

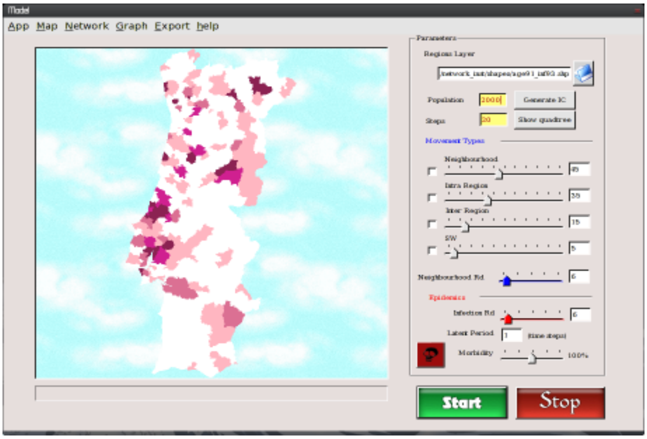

**FIGURE 11.** Initial Conditions for the simulations.

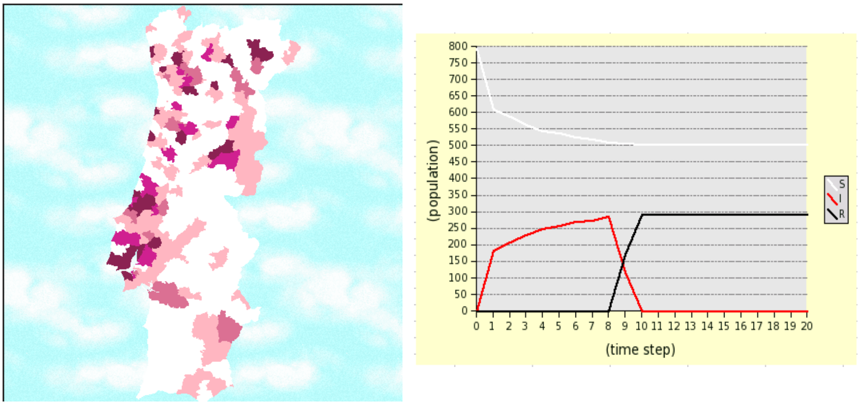

**FIGURE 12.** Simulation using neighbourhood movement only.

In the neighbourhood movement simulation (Fig. 12), the infection is much more restricted, in magnitude and in spatial extension, than in all other simulations. This is obviously due to the tighter movement range.

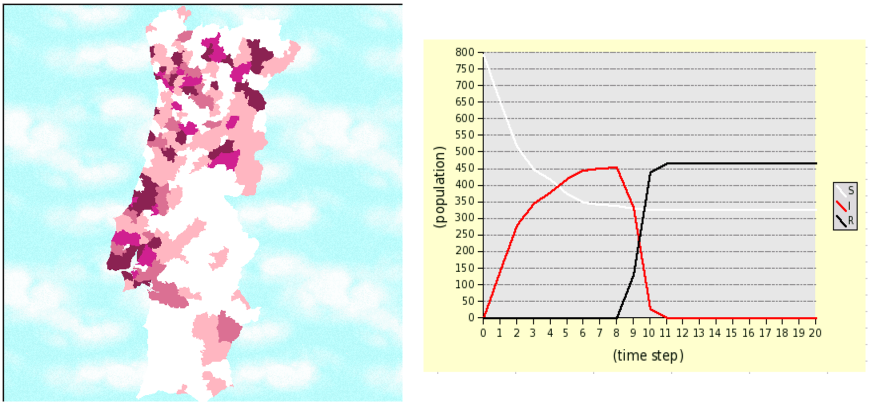

**FIGURE 13.** Simulation using intra region movement only.

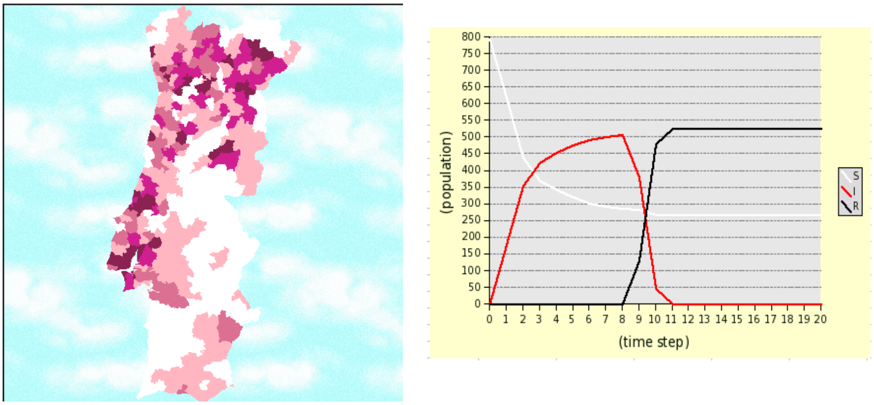

**FIGURE 14.** Simulation using inter region movement only.

It is also important to remark that the stability of the epidemic, occurs earlier in the neighbourhood simulations than in all others.

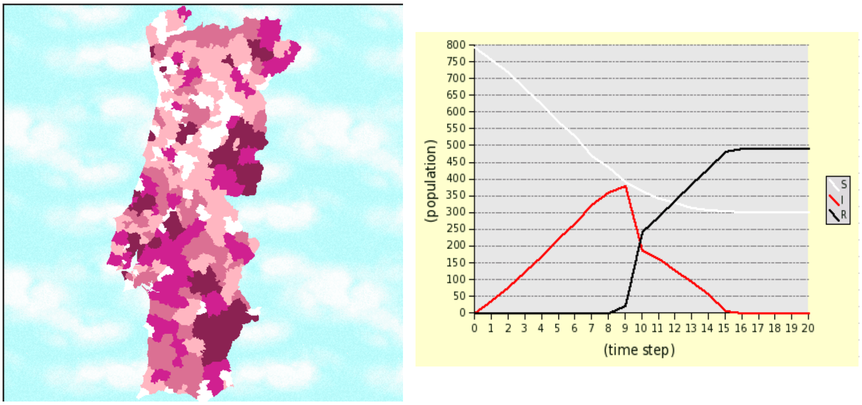

**FIGURE 15.** Simulation using Small World movement only.

The Small World movement simulation (Fig. 15) presents a totally different distribution of population. As the individuals reach every part of the domain, so does the epidemic.

However, for being so contagious its inefficiency is reduced because many individuals die before they transmitted the disease, and so the stabilization of the epidemic is reached later than in the other simulations.

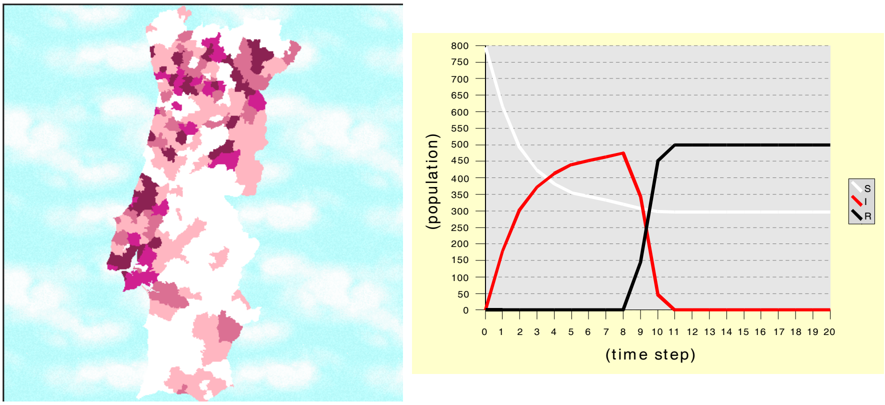

**FIGURE 16.** Simulation using all components of movement but Small Worlds.

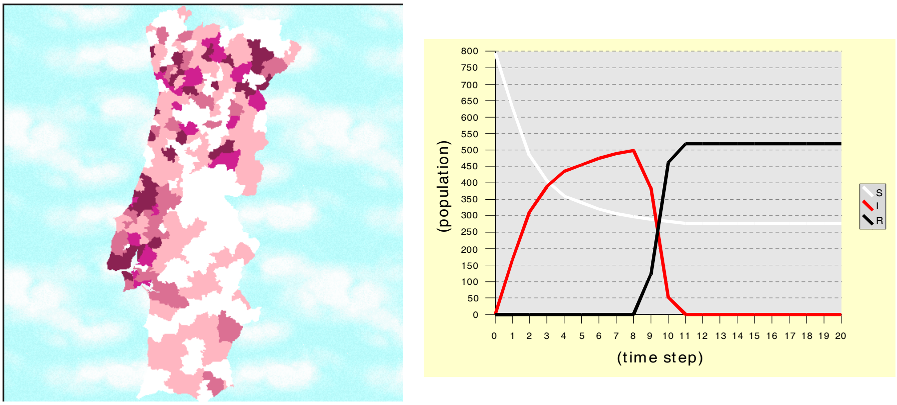

**FIGURE 17.** Simulations using all components of movement, in the proportions suggested on Fig. 3.

The impact of this component, even if present in a small amount, can be seen by running a simulation with no small world movement (Fig. 16) and another simulation with a small world movement probability of 0.05 (Fig. 17).

In the case when the Small World movement is included, the epidemic reaches a greater number of people, and reaches a greater part of the country.

This analysis calls attention to the importance of the mobility network, embedded in the epidemic model as it has a determinant impact in the evolution of the epidemic.

In this network, it was shown how the small component of random movement (characteristic of Small World networks) has an effective influence on the results, which enforces the belief that it should not be ignored when modelling social networks.

However, by now this study still lacks of a network analysis, observing measures such as the average path length and clustering coefficient, that will allow to evaluate if a small world network has effectively emerged.

### CONCLUSIONS AND FUTURE DEVELOPMENTS

As it was demonstrated on the previous chapter, the structure of the mobility network is determinant in the spatial pattern and on the magnitude of the epidemic. The movement should always be considered in human epidemics models.

Another conclusion from this work is that the Agent based approach is very well suited for epidemic modelling and that vector based modelling , with a appropriated programming, is quite efficient and provides a realistic representation of reality.

However there is still a lot of work to be done: the movement model needs to be analysed in terms of networks, and the infection model needs to be tested, to evaluate the importance of the different parameters and, in the future, fit the characteristics of specific diseases.

There is already one dataset introduced in the model and some data analysis needs to be conducted to evaluate the model efficacy. Although, due to several issues, it is always is difficult to match the model results with real data, this would provide a way of validate it.

Finally, one of the most useful applications of a spatial model like this, will be the introduction of vaccination barriers, that will allow to study different vaccination strategies.

### ACKNOWLEDGMENTS

I would like to thank my supervisor, Michael Batty (CASA) for reviewing the presentation that originated this paper and Carmo Gomes (FCUL) for providing me the dataset I use on these simulations.
